# The Long Noncoding RNA Expression Profile of Hepatocellular Carcinoma Identified by Microarray Analysis

**DOI:** 10.1371/journal.pone.0101707

**Published:** 2014-07-15

**Authors:** Juanjuan Zhu, Shanshan Liu, Fuqiang Ye, Yuan Shen, Yi Tie, Jie Zhu, Yinghua Jin, Xiaofei Zheng, Yongge Wu, Hanjiang Fu

**Affiliations:** 1 College of Life Sciences, Jilin University, Changchun, China; 2 Beijing Institute of Radiation Medicine, Beijing, China; CSIR Institute of Genomics and Integrative Biology, India

## Abstract

**Background:**

Thousands of long noncoding RNAs (lncRNAs) have been reported in mammalian genomes. These RNAs represent an important subset of pervasive genes involved in a broad range of biological functions. Aberrant expression of lncRNAs is associated with many types of cancers. Here, in order to explore the potential lncRNAs involved in hepatocellular carcinoma (HCC) oncogenesis, we performed lncRNA gene expression profile analysis in 3 pairs of human HCC and adjacent non-tumor (NT) tissues by microarray.

**Methodology:**

Differentially expressed lncRNAs and mRNAs were detected by human lncRNA microarray containing 33,045 lncRNAs and 30,215 coding transcripts. Bioinformatic analyses (gene ontology, pathway and network analysis) were applied for further study of these differentially expressed mRNAs. By qRT-PCR analysis in nineteen pairs of HCC and adjacent normal tissues, we found that eight lncRNAs were aberrantly expressed in HCC compared with adjacent NT tissues, which is consistent with microarray data.

**Conclusions:**

We identified 214 lncRNAs and 338 mRNAs abnormally expressed in all three HCC tissues (Fold Change ≥2.0, P<0.05 and FDR <0.05) with the genome-wide lncRNAs and mRNAs expression profile analysis. The lncRNA-mRNA co-expression network was constructed, which may be used for predicting target genes of lncRNAs. Furthermore, we demonstrated for the first time that BC017743, ENST00000395084, NR_026591, NR_015378 and NR_024284 were up-regulated, whereas NR_027151, AK056988 and uc003yqb.1 were down-regulated in nineteen pairs of HCC samples compared with adjacent NT samples. Expression of seven lncRNAs was significantly correlated to their nearby coding genes. In conclusion, our results indicated that the lncRNA expression profile in HCC was significantly changed, and we identified a series of new hepatocarcinoma associated lncRNAs. These results provide important insights about the lncRNAs in HCC pathogenesis.

## Introduction

The incidence of hepatocelluar carcinoma is currently the fifth highest in solid tumor and is the third leading cause of cancer death worldwide, accounting for approximately one million deaths annually [1], [2]. Geographic areas with highest frequency are located in Africa and Eastern Asia. Most cases of HCC are attributed to either chronic viral hepatitis infection (hepatitis B or C) or cirrhosis [3]. Unfortunately, the survival rate of HCC patients remains poor despite recent advances in medical treatment and surgical techniques [4]. More seriously, the mortality of HCC is still growing with an increasing trend of new occurrence [4].

The overwhelming majority of human transcriptome was confirmed to be noncoding genes [5]. Over the past decade, those abundant transcripts have been declared to have important regulatory potential in biological processes. Increasing evidence have pointed to the critical regulatory role of noncoding RNAs (ncRNAs) in normal cellular physiological processes as well as the contribution of aberrant ncRNA expression to cancer biology [6]. According to their length, ncRNAs can be divided into two major categories, long noncoding RNAs which are tentatively defined as a class of RNA molecules longer than 200 nucleotides and short noncoding RNAs (18 to 200 nucleotides) respectively [7]. It is well documented that microRNAs are aberrantly expressed in many types of cancer. MicroRNA-21, microRNA-122 and microRNA-657 are involved in the development of HCC [8]–[11]. Recently, lncRNAs have been shown to exert critical roles in a series of biological processes, including genetic imprinting, immune response, tumorigenesis, cellular development and metabolism through comprehensive mechanisms [5], [12]. LncRNAs also played critical roles in a variety of human diseases, including cancer, Huntington's disease and Alzheimer's disease [13]–[15]. Deregulation of lncRNAs has been proposed to be associated with hepatocarcinogenesis, such as MVIH, H19, HEIH, HULC, TUC338 and MEG3 [16]–[22]. For example, lncRNA high expression in HCC (lncRNA-HEIH) facilitates tumor growth through Enhancer of Zeste Homolog 2 in humans [18]; LncRNA-HULC which is a highly specific up-regulated lncRNA in HCC, envisaged as a novel biomarker because it could be detected in blood of HCC patients [19]; The expression of LncRNA-MALAT-1 up-regulated predicts tumor recurrence of HCC after liver transplantation [20]. Despite these exciting development, many more lncRNAs playing crucial roles in HCC remain to be clarified.

**Figure 1:**
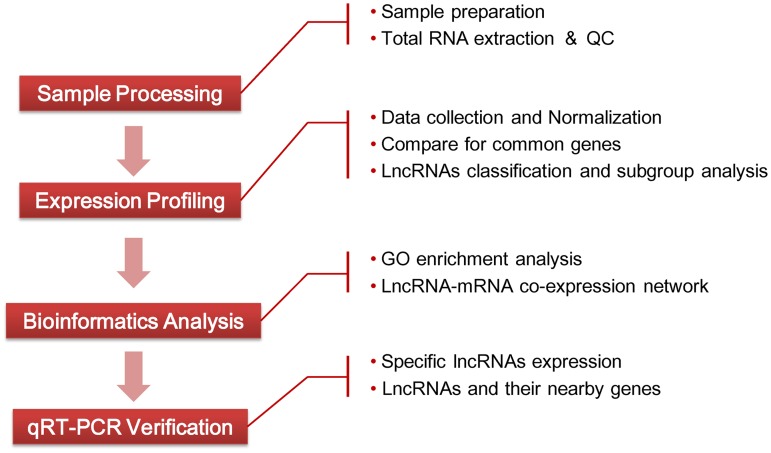
Flowchart of the experiment.

By analyzing the global expression profile of lncRNAs and mRNAs, we identified 214 lncRNAs and 338 mRNAs that were significantly differentially expressed in HCC tissues and adjacent NT tissues. LncRNA classification and subgroup analysis, genomic location analysis, construction of the lncRNA-mRNA co-expression network and qRT-PCR analysis were performed for further analyze these differentially expressed lncRNAs (Figure 1). We found eight lncRNAs were dysregulated in nineteen pairs of HCC samples compared with adjacent NT samples and expression of seven lncRNAs was significantly correlated to their nearby coding genes. Simultaneously, the lncRNA-mRNA co-expression network was constructed, which may be used for predicting target genes of lncRNAs. Our observations demonstrated that lncRNAs expression profiles are likely to provide important insights into pathogenesis of HCC.

## Materials and Methods

### Patient sample preparation

Nineteen pairs of HCC samples and adjacent non-tumor tissues were obtained from surgical specimens at General Hospital of the People's Liberation Army (Beijing, China) after informed consent. Those samples used in this study have been described in previous publication [23]. Clear hepatocellular carcinoma was diagnosed histopathologically. All these specimens including clear hepatocellular carcinoma and adjacent non-tumor tissues were snap-frozen at liquid nitrogen after excision. Of these samples, 3 pairs (2 patients infected with Hepatitis B, and one infected with Hepatitis C) were used for lncRNA microarray analysis and the remaining samples were used for further validation. The clinical characteristics of the three HCC patients were shown in Table 1.

**Table 1:** Clinical Characteristics of the Patients.

Patient No.	Age	Gender	Tumer Size (cm*cm*cm)	HBsAg	HCV-Ab	Cirrhosis
H1	37	M	16*4*8	Positive	Negative	Yes
H2	64	M	8*5*4	Negative	Positive	No
H3	52	M	7*7*7	Positive	Negative	Yes

HBsAg indicates hepatitis B surface antigen; HCV-Ab, hepatitis C virus antibody; M, male.

### RNA extraction

Total RNA was extracted from the frozen tissue block using TRIzol reagent (Sigma, USA) according to the manufacturer's protocol. RNA quantification and quality were assured by Nano Drop ND-1000 spectrophotometer. RNA integrity and gDNA contamination were tested by denatured agarose gel electrophoresis (Figure S1).

### Microarray analysis and computational analysis

Sample preparation and microarray hybridization were performed by Kangchen Bio-tech, Shanghai P.R. China. Briefly, RNA was purified from 1 µg total RNA after removal of rRNA (mRNA-ONLY Eukaryotic mRNA Isolation Kit, Epicentre). Then, each sample was amplified and transcribed into fluorescent cRNA along the entire length of the transcripts without 3′ bias utilizing a random priming method. The labeled cRNAs were hybridized onto the Human LncRNA Array v2.0 (8×60 K, Arraystar). After having washed the slides, the arrays were scanned by the Agilent Scanner G2505B. Agilent Feature Extraction software (version 10.7.3.1) was utilized to analyze acquired array images. Quantile normalization and subsequent data processing were carried out using the GeneSpring GX v11.5.1 software package (Agilent Technologies). Differentially expressed LncRNAs and mRNAs were identified through fold change filtering (Fold Change ≥2.0 or ≤0.5), paired t-test (p<0.05) and multiple hypothesis testing (FDR<0.05). P values and FDR were calculated by Microsoft Excel and MATLAB respectively. The microarray data has been deposited in NCBI Gene Expression Omnibus (GEO) and the GEO accession number is GSE55191 (http://www.ncbi.nlm.nih.gov/geo/query/acc.cgi?acc=GSE55191). Pathway analysis and GO analysis were applied to determine the roles of these differentially expressed mRNAs played in these biological pathways or GO terms. Differentially regulated mRNAs were uploaded into the Database for Annotation, Visualization and Integrated Discovery (DAVID, http://david.abcc.ncifcrf.gov/) to analyze the enrichment of these coding genes. After uploaded 338 seqnames of differentially regulated mRNAs into Functional Annotation Chart, 325 mRNAs were matched to the list. The annotation summary results were shown up by this webserver.

### Quantitative real-time polymerase chain reaction (qRT-PCR) validation and Statistical analysis

Total RNA was reverse-transcribed using ImProm II Reverse Transcriptase (Promega) according to the manufacturer's protocol. Real-time PCR was done with SYBR Premix Ex Taq (TaKaRa) on MX3000p instrument according to the manufacturer's protocol. Specific primers of each genes are listed in Table S1. The relative fold change was calculated using the 2^−ΔΔCt^ method normalized to GAPDH. Differences of lncRNAs between tumor and normal were analyzed using paired t-test. P value <0.05 was considered as statistically significant.

### Co-expression network construction

The lncRNA-mRNA co-expression network was constructed based on the correlation between the differentially expressed lncRNAs and mRNAs. The algorithm was quoted from previously described report [24]. Red ellipse represents as down-regulated lncRNA, green diamond represents as down-regulated mRNA, blue ellipse represents as up-regulated lncRNA, black diamond represents as up-regulated mRNA. Solid lines represent positive correlation, dotted lines represent negative correlation.

## Results

### Expression profile of lncRNAs and mRNAs in HCC

Fold-change (Tumor vs Normal) and *P* value were calculated from the normalized expression. Using microarray analysis, we identified 214 lncRNAs and 338 mRNAs to be significantly differentially expressed in three HCC samples compared with their adjacent NT samples (Fold Change ≥2.0 or ≤0.5, p<0.05 and FDR<0.05). Among these, 166 lncRNAs and 219 mRNAs were identified to be consistently upregulated in all three HCC groups, and 48 lncRNAs and 119 mRNAs were consistently downregulated (Table S2). The expression of ENST00000448647 (Log2 Fold Change T/N = 7.8561822) was significantly upregulated, while the expression of ENST00000504368 (Log2 Fold Change T/N = −7.4821358) was dramatically downregulated. The number of deregulated lncRNAs and mRNAs varied in different patients (Table 2, 3). These lncRNAs may play important roles in the occurrence and development of HCC.

**Table 2:** Number of aberrantly expressed lncRNAs in microarray for three pairs of HCC and adjacent non-tumor tissues.

Sample ID		Long noncoding RNA				
		Fold change 2–4	Fold change 4–6	Fold change >6	Total	Aberrant lncRNAs
H1	up	1917	461	335	2713	4369
	down	1120	235	301	1656	
H2	up	1393	301	240	1934	3312
	down	1033	177	168	1378	
H3	up	1314	665	1901	3880	7093
	down	1898	628	687	3213	

**Table 3:** Number of aberrantly expressed mRNAs in microarray for three pairs of HCC and adjacent non-tumor tissues.

Sample ID		Message RNA				
		Fold change 2–4	Fold change 4–6	Fold change >6	Total	Changed mRNAs
H1	up	2295	487	428	3210	6077
	down	1852	419	596	2867	
H2	up	2001	380	326	2707	5132
	down	1595	381	449	2425	
H3	up	1831	918	2402	5151	10110
	down	2709	885	1365	4959	

### LncRNA Classification and Subgroup Analysis

According to previous reports, lncRNAs can be classified into different subgroups, such as HOX lncRNAs, lncRNAs with enhancer-like function (lncRNA-a) and large intergenic noncoding RNAs (lincRNAs) [25], [26]. We carried out lncRNA classification and subgroup analysis to further investigate potential functions of these HCC associated lncRNAs.

Rinn *et al.* have identified a total of 407 discrete lncRNAs within the four human homeobox transcription factors (HOX) clusters, partitioned into 101 known HOX gene exons, 75 introns and 231 intergenic transcripts [26]. Among them, 6 of coding transcripts and 4 of noncoding transcripts were differentially expressed in three sets of HCC samples (Table S3).

Using the GENCODE annotation [27] of the human genome, Orom *et al.* have defined a set of lncRNAs with enhancer-like function in human cell lines [25]. Depletion of these lncRNAs led to decreasing expression of their neighboring protein-coding genes. The profiling data showed that 506 such lncRNAs were detected, while 26 of them were differentially expressed. Among these 26 lncRNAs, 15 were located in nearby aberrantly expressed coding genes (distance <300 kb) (Table S4, S5), implying that these lncRNAs might be regulators of their neighboring coding genes in HCC cells.

By analyzing chromatin-state maps, Rinn and his colleagues have identified ∼3,300 lincRNAs with clear evolutionary conservation and association with diverse biological processes, including cell proliferation, immune surveillance, cell-cycle regulation and embryonic stem cell pluripotency [28], [29]. The profiling data indicated that 46 of such lincRNAs were dysregulated in HCC samples, and 35 among them were located in nearby aberrantly expressed coding genes (distance <300 kb) (Table S6, S7).

### Bioinformatic analysis of mRNAs expression in HCC

**Figure 2:**
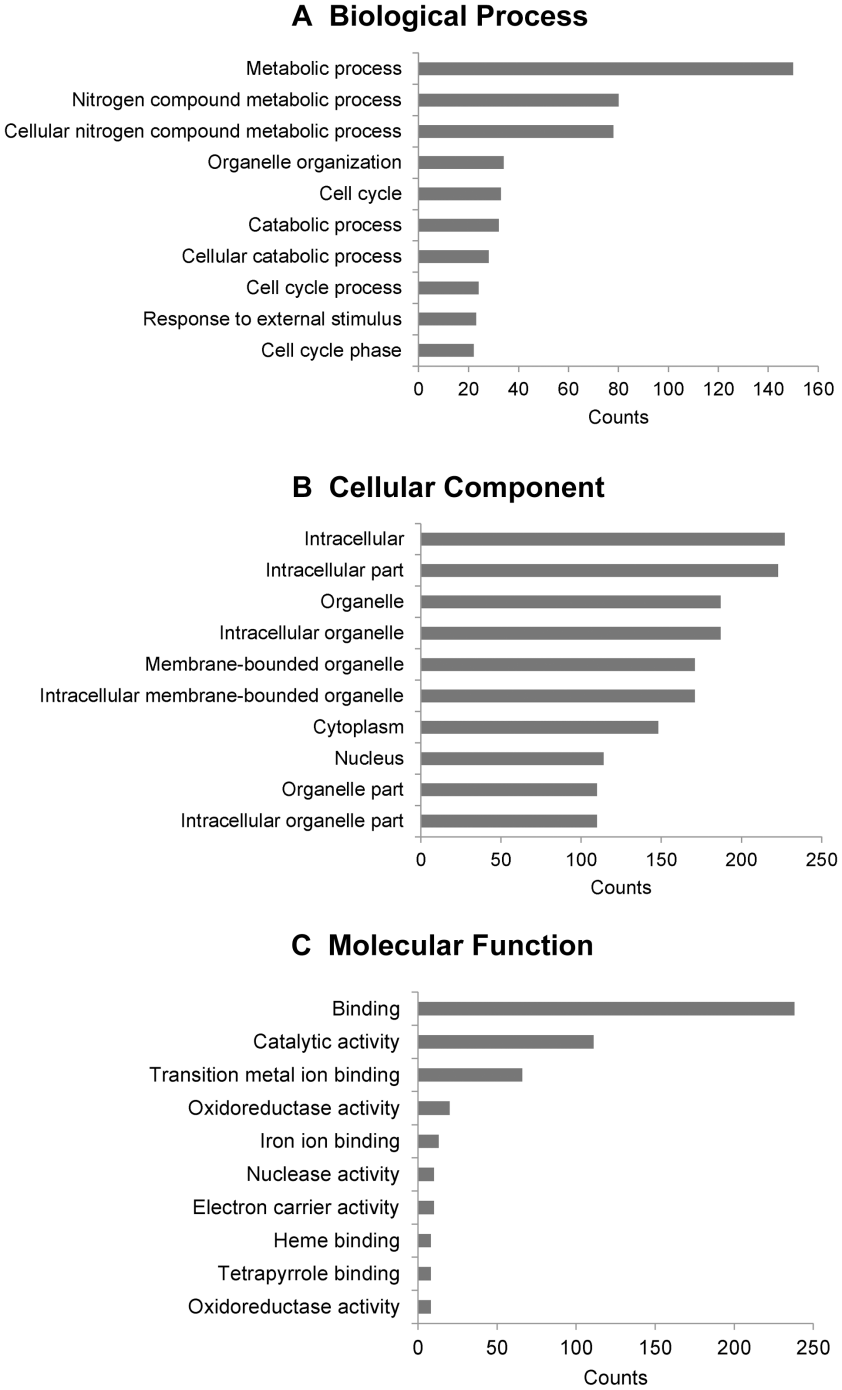
Gene ontology (GO) enrichment analysis for differentially regulated mRNAs. (A) GO analysis of mRNAs according to biological process. (B) GO analysis of mRNAs according to cellular component. (C) GO analysis of mRNAs according to molecular function.

Up to 12,751 coding transcripts could be detected in three pairs of samples through 30,215 coding transcripts probes. 338 mRNAs were differentially expressed in all three pairs of samples. Using DAVID Functional Annotation Chart, we analyzed the enrichment of these 338 differentially regulated mRNAs (Table S8). The result showed that the most significant functional groups consisted of metabolic process, cellular component organization, cell cycle and response to chemical stimulus (Figure 2). Liver is a highly metabolic organ balancing various biological processes. Its disorder is an important cause for the development and progression of HCC [30]. Specific nutrients or metabolites are associated with increased HCC risk. Recent data have suggested that several metabolic regulatory drugs may have the potential to decrease the incidence of HCC [31], [32].

### Genomic location of these differentially expressed lncRNAs

The functional characterization of these lncRNAs presents a formidable task, and some evidences suggested that lncRNAs regualte high order chromosomal dynamics, sub-cellular structural organization and telomere biology [33]. LncRNAs have been dedicated to originate from complex loci that include lncRNAs and associated protein-coding genes. For example, a number of lncRNAs have been reported to regulate the expression of adjacent protein-coding genes [34], [35]. The relationship of lncRNAs and its nearby coding gene identified here includes bidirectional, exon sense-overlapping, intergenic, intro sense-overlapping, intronic antisense and natural antisense. Among the 214 consistently regulated lncRNAs in three sets of HCC samples, including 29 directional sequences (23 upregulated and 6 downregulated), 18 exon sense-overlapping sequences (16 upregulated and 2 downregulated), 120 intergenic sequences (93 upregulated and 27 downregulated), 4 intro sense-overlapping sequences (3 upregulated and 1 down regulated), 29 inronic antisense (19 upregulated and 10 downregulated) and 27 natural antisense (23 upregulated and 4 downregulated). Such information about these lncRNAs and their nearby coding genes might be useful to predict their functional roles in HCC (Table S2).

### Construction of the lncRNA-mRNA Co-expression Network

In order to ascertain the correlation between differentially expressed lncRNA and mRNA in HCC, we constructed a lncRNA-mRNA co-expression network as described [24]. Pearson correlation coefficients between all aberrant lncRNAs and mRNAs were calculated. Using P-value <0.001 and absolute value of correlation coefficient ≥0.99, we identified 249 pairs of co-expressed lncRNAs and mRNAs composed of 131 mRNAs (39% of all differentially expressed mRNAs) and 103 lncRNAs (48% of all differentially expressed lncRNAs), and 146 pairs presented as positive correlation (Figure S2). LncRNA-mRNA pairs with the highest positive correlation coefficiency included BC016002-ZNF777, ENST00000424412-TUBB2A and CR625009-RPL17. ENST00000444546-CES1 and ENST00000433673, uc.472-PLG had the highest negative correlation coefficiency. This network indicated that one lncRNA could correlate with one to tens of mRNAs and so was the mRNA. Increasing evidences indicated that lncRNAs play roles in gene expression [36]. For example, long intergenic non-coding RNA-p21 activated by p53 mediates global gene repression in the p53 response [37]. Thus, we proposed that the expression profile of mRNAs and lncRNAs are significantly correlated.

### A set of lncRNAs were dysregulated in HCC samples

**Figure 3:**
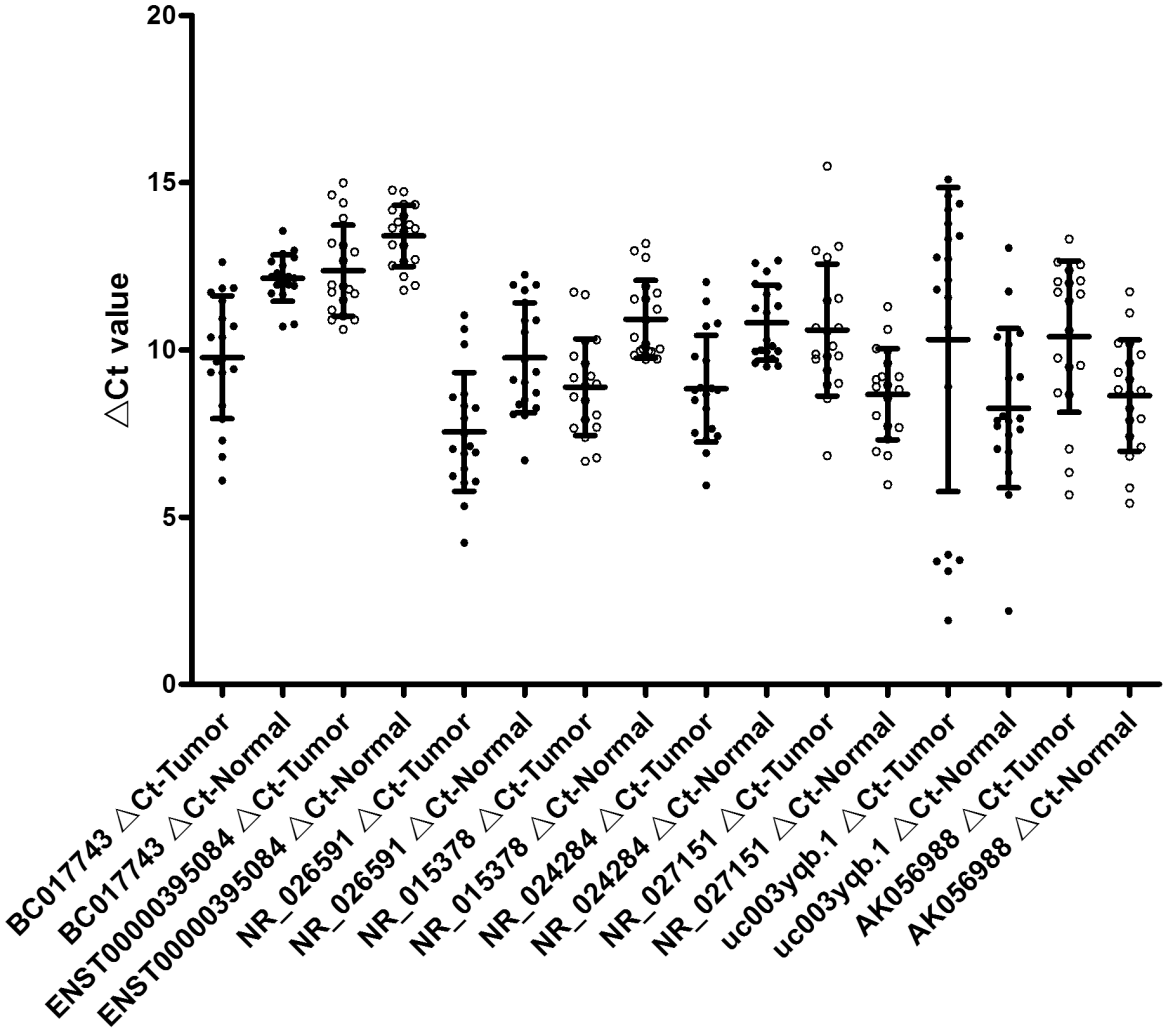
Distribution of eight lncRNAs in nineteen pairs of HCC samples. Selected eight differentially expressed lncRNAs were validated by qPCR in 19 pairs of HCC samples. The heights of the columns in the chart represent the relative concentration of each lncRNA validated across the 19 patients.

**Figure 4:**
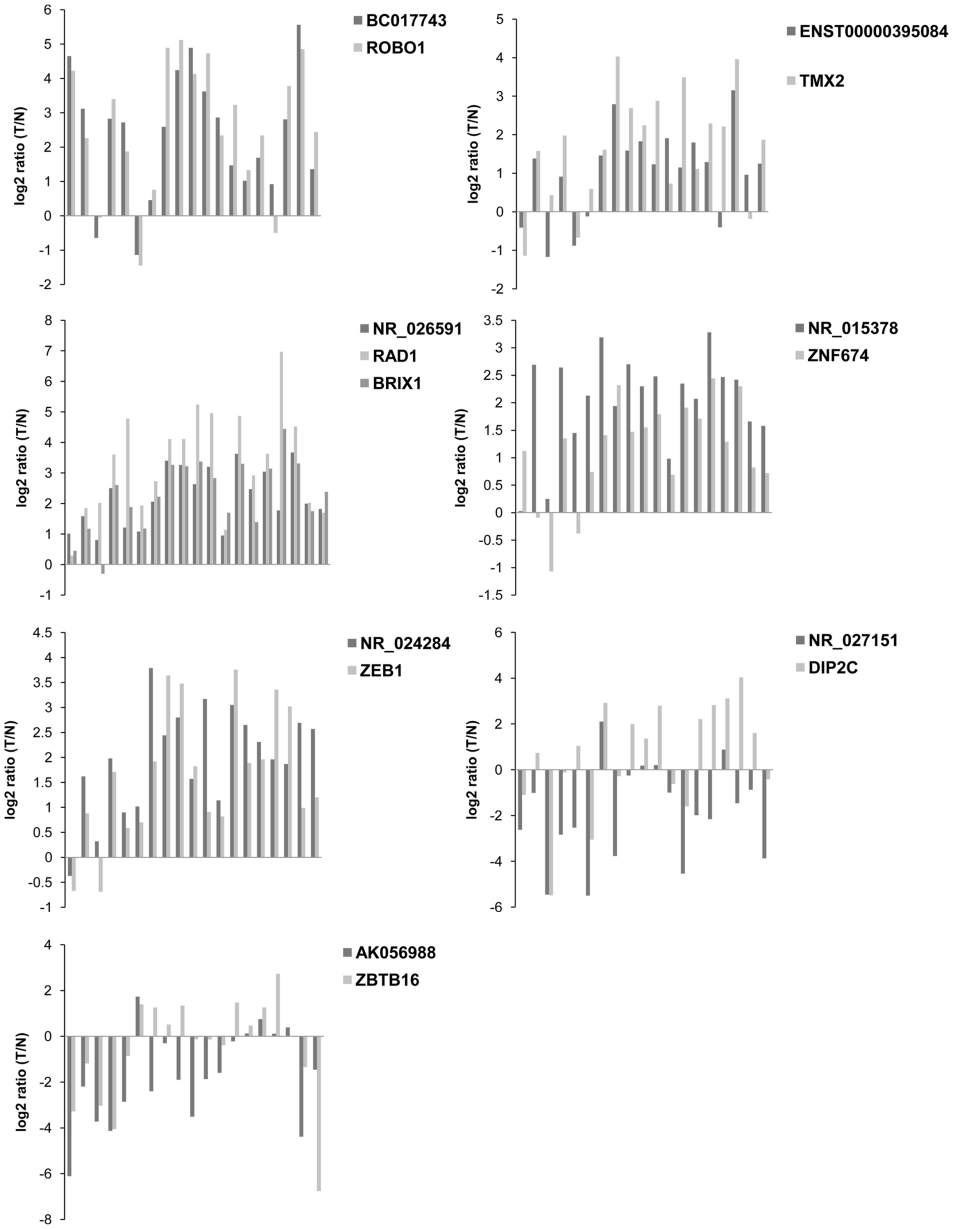
Consistency of lncRNAs and their nearby genes expression level. Selected seven differentially expressed lncRNAs and their nearby genes were validated by qPCR in 19 pairs of HCC samples. The heights of the columns in the chart represent the log2 fold changes (T/N) in expression across the 19 patients for each of the seven lncRNAs validated.

To verify the microarray data, we randomly selected fifty differentially expressed lncRNAs including twenty up-regulated lncRNAs and thirty down-regulated ones and validated their expression levels by quantitative RT-PCR (qRT-PCR) in three sets of HCC tissues. The result showed that the expression patterns of forty lncRNAs were consistent with the microarray data (Table 4). Subsequently, eight lncRNAs from these forty were evaluated in the nineteen pairs of HCC and adjacent NT samples using qRT-PCR (Table S9). The results showed that BC017743, ENST00000395084, NR_026591, NR_015378 and NR_024284 were up-regulated and NR_027151, AK056988 and uc003yqb.1 were down-regulated in HCC samples compared with adjacent NT samples (Figure 3). Additionally, microarray data also provided the nearby coding gene of these differentially expressed lncRNAs (Table S10). We found that the changing trend of lncRNAs and their nearby coding genes is basically identical through qRT-PCR analysis of a selected number of differentially expressed lncRNAs and their nearby genes (Figure 4). These results provided strong evidence for the point that lncRNAs have intrinsic cis-regulatory capacity to their own locus.

**Table 4:** Quantitative RT-PCR confirmation for selected forty lncRNAs.

Seqname	Chromosome	ncRNAtype	Microarray log2 ratio(T/N)			RT-qPCR log2 ratio(T/N)		
			H1	H2	H3	H1	H2	H3
AK056250	chr1	intergenic	2.86	2.39	1.85	1.16	0.96	1.03
BC013423	chr5	intergenic	2.62	2.62	4.93	1.49	1.50	2.90
BC017743	chr3	intron sense-overlapping	5.41	1.31	3.54	3.81	0.20	0.04
ENST00000394293	chr7	intergenic	1.20	2.07	1.34	0.19	0.99	0.61
ENST00000417262	chr1	intergenic	2.74	1.60	3.01	2.29	0.87	0.75
NR_002734	chr8	intronic antisense	3.64	2.13	3.19	3.18	1.71	3.23
NR_003573	chr9	intergenic	2.50	1.21	2.96	2.16	1.26	1.49
uc001vvq.1	chr14	intergenic	1.75	3.70	5.79	0.16	0.86	1.04
uc003gzp.2	chr4	intergenic	3.23	1.03	2.56	2.59	0.90	0.86
ENST00000447956	chr20	intergenic	3.73	4.03	2.91	2.83	3.26	1.17
NR_027355	chr1	intergenic	1.95	1.15	3.59	1.00	0.86	3.24
uc001tvk.1	chr12	intergenic	4.92	4.03	4.30	2.87	0.78	3.69
uc002odt.1	chr19	intergenic	4.84	2.22	5.36	4.64	0.97	0.93
uc001sqo.2	chr12	intergenic	1.50	3.21	4.68	2.06	1.58	1.54
ENST00000421424	chr20	intergenic	−6.38	−2.73	−1.96	−5.50	−2.14	−2.32
ENST00000423174	chr3	intergenic	−2.84	−1.84	−5.87	−1.79	−2.16	−1.94
ENST00000502804	chr4	intergenic	−4.28	−2.75	−1.95	−3.22	−4.00	−1.67
ENST00000504368	chr4	intergenic	−1.90	−2.77	−7.48	−2.75	−2.16	−9.42
X91348	chr22	intergenic	−3.68	−2.92	−4.39	−2.83	−2.37	−0.33
ENST00000449772	chr2	intergenic	−2.59	−1.99	−2.59	−3.54	−4.10	−5.74
ENST00000437516	chr6	intergenic	−9.53	−1.02	−1.42	−0.51	−0.23	−2.84
ENST00000418694	chr9	intergenic	−2.87	−1.43	−3.22	−1.24	−0.79	−2.20
ENST00000451163	chr2	intergenic	−3.26	−7.76	−5.81	−1.45	−2.12	−2.40
ENST00000489312	chr4	natural antisense	−6.79	−1.18	−1.87	0.07	0.30	−2.66
NR_033646	chr16	exon sense-overlapping	−3.66	−1.15	−6.74	−1.26	−1.38	−2.51
uc010ytt.1	chr2	intergenic	−2.96	−7.66	−7.14	−3.75	−8.17	−6.38
AK124428	chr5	intergenic	−2.01	−1.48	−1.44	−2.45	−1.54	−1.24
AK129699	chr7	intergenic	−1.81	−2.35	−2.84	−2.14	−2.57	−3.52
ENST00000377415	chr9	intergenic	−1.68	−4.91	−4.89	−1.61	−5.20	−5.37
ENST00000398087	chr7	intergenic	−2.92	−2.12	−6.30	−3.68	−1.09	−7.82
NR_002712	chr2	intergenic	−4.32	−3.74	−1.91	−2.90	−3.49	−4.97
NR_026643	chr11	intronic antisense	−2.99	−1.04	−5.62	−4.08	−1.06	−7.73
NR_026743	chr10	intergenic	−3.29	−2.07	−4.09	−5.13	−1.22	−9.23
NR_027151	chr10	intronic antisense	−3.69	−5.79	−5.69	−2.83	−3.11	−5.63
NR_033661	chr22	exon sense-overlapping	−2.18	−4.55	−4.42	3.05	−5.67	−3.63
ENST00000416474	chr2	intergenic	−3.61	−7.65	−4.93	−2.17	−0.74	−0.61
ENST00000417888	chr10	intergenic	−2.56	−3.43	−1.97	1.88	−1.31	−3.18
AK056988	chr11	intronic antisense	−4.15	−1.32	−2.99	−5.39	−2.34	−3.82
U70031	chr2	intergenic	−3.40	−8.10	−3.68	−2.47	−5.72	−5.39
uc003yqb.1	chr8	intronic antisense	−3.92	−1.09	−5.34	−6.66	−2.80	−6.97

## Discussion

HCC is the fifth most common malignancy in human beings, accounting for approximately 90% of primary liver cancers [38]. Hepatocarcinogenesis is a complicated biological process characterized by a myriad spectrum of molecular abnormalities. Over the past decades, the molecular mechanism of HCC has been extensively investigated. However, the exact pathogenesis of this disease is still vague. Increasing evidence indicates that lncRNAs may play a significant role in regulating gene expression [39]. LncRNA expression is de-regulated in many types of cancers, such as HOTAIR in breast tumours and metastases, BACE1-AS in Alzheimer's disease and Gas5 in breast cancer [15], [40], [41]. Dysregulation of lncRNAs, including H19, HEIH, MVIH, HULC and MEG3, has been identified in HCC [16]–[18], [22]. Some of these lncRNAs were also identified in our microarry data. For example, H19 was downregulated in H1 and H2 tissues. Therefore, it is a critical step to figure out the expression profile of lncRNAs and related mRNAs in HCC in understanding its pathogenesis of HCC.

In this study, we investigated gene expression profiles of HCC by using lncRNA microarray. Compared with previous report [18], which showed hierarchical clustering analysis of 254 mRNAs and 174 lncRNAs that were differentially expressed between five pairs of HCC samples and nontumor samples (all samples using here are HBV-related), we analyzed three pairs of HCC and adjacent non-tumor samples (two patients infected with Hepatitis B, and one infected with Hepatitis C) and identified 214 lncRNAs and 338 mRNAs abnormally expressed in all three HCC tissues (Fold Change ≥2.0, P<0.05 and FDR<0.05) which is partly different from previous results. This is due to differences in cancerous tissues. Moreover, the microarray which we used is human lncRNA microarray version 2.0, whereas the microarray which was used in the above report is human lncRNA microarray version 1.0. Compared with version 1.0, version 2.0 contains more comprehensive and reliable array content, most extensive and updated coverage available, specific exon or splice junction probes, efficient and robust labeling system and systematic lncRNA classification. According to the absolute expression profile in three pairs of sample, the numbers of significantly differentially expressed lncRNAs and mRNAs (Fold Change ≥2.0 or ≤0.5) are 624 and 1050 separately. After statistical analysis (the threshold for deregulated genes was p<0.05 and FDR<0.05), this number decreased to only 214 and 338. This phenomenon might be because the gene expression level varies considerably in different patients. Therefore, this statistical analysis will lead to the results that many important differentially expressed genes have been excluded.

Yang F *et al.* have confirmed that AY129027, uc002pyc, and DQ786243 were overexpressed, whereas the expression of AK055007 and AK123790 was decreased in 50 paired HCC compared with adjacent NT samples using RT-PCR [18]. In our study, we demonstrated that BC017743, ENST00000395084, NR_026591, NR_015378 and NR_024284 were up-regulated, whereas NR_027151, AK056988 and uc003yqb.1 were down-regulated in nineteen pairs of HCC samples compared with adjacent NT samples using qRT-PCR. Moreover, expression of seven lncRNAs was significantly correlated with their nearby coding genes which have not been described before. For example, NR_026591 is a 4525 bp exon sense-overlapping lncRNA transcribed from chr5. It is one of the alternatively spliced transcript variants of the RAD1 gene, which encodes a component of the 9-1-1 complex, a heterotrimeric cell cycle checkpoint complex involved in stopping cell cycle progression. NR_026591 and BRIX1 make up a bidirectional transcript pair, expression of which are initiated in opposite directions and in close proximity [42]. QRT-PCR results showed that the expression trend of protein-coding genes (RAD1, BRIX1) and lncRNA (NR_026591) was consistent. We speculate that NR_026591 may have cis-regulatory capacity to their own locus. All the seven lncRNAs (Table S10) are uncharacteristic and associated biological functions of them are unclear. The present data will be beneficial to more in-depth exploration of HCC.

LncRNAs are involved in transcriptional regulation of many protein-coding genes. In order to obtain novel insight into the function of lncRNAs, we further combined differentially expressed lncRNAs with differentially expressed mRNAs to construct a co-expression network. Owing to differences of abnormally expressed lncRNAs and mRNAs, the lncRNA-mRNA co-expresion network is different from the network described in the previous report. We found that many lncRNAs were significantly correlated with multiple protein-coding genes. For example, this network indicated that ZNF777 was associated with BC016002. BC016002 is a 960 bp intergenic lncRNA located on chr7. ZNF777 is involved in transcriptional regulation due to its DNA binding function which is the most significant functional group in the result of GO analysis of differentially regulated mRNAs. Therefore, we hypothesized that ZNF777 may be the direct or indirect target genes of BC016002. This network also indicated that CES1 are predicted to be associated with ENST00000444546. ENST00000444546 is a 1150 bp intergenic lncRNA located on chr1. CES1 encodes a member of the carboxylesterase large family which are responsible for the hydrolysis or transesterification of various xenobiotics. This enzyme is the major liver enzyme and functions in liver drug clearance. Drug clearance which is an important characteristic of CES1 may be critical for the function of ENST00000444546. ENST00000433673 is a 2690 bp intergenic lncRNAs located on chr2 and uc.472 is a 202 bp exon sense-overlapping lncRNA located on chrX. Their correlated gene in co-expression network is PLG. Protein encodes by this gene is plasminogen which is activated by proteolysis and converts to plasmin and angiostatin. Plasmin is an essential protease in a variety of cellular processes including embryonic development, tissue remodeling, tumor invasion and inflammation. Angiostatin is an angiogenesis inhibitor that blocks neovascularization and growth of primary and metastatic tumors. This characteristic indicates that ENST00000433673 and uc.472 may be associate with PLG to exert its function in HCC. The lncRNA and mRNA co-expression network provides a strong foundation for predicting the function of lncRNA. The malfunction of regulation in this co-expression network may be a possible step for development and progression of HCC. The precise molecular mechanisms have yet to be defined.

In conclusion, we described the expression profile of lncRNAs and mRNAs in HCC by microarray and identified 214 lncRNAs and 338 mRNAs abnormally expressed in all three HCC tissues (Fold Change ≥2.0, P<0.05 and FDR<0.05). After qRT-PCR verification, we demonstrated for the first time that BC017743, ENST00000395084, NR_026591, NR_015378 and NR_024284 were up-regulated, whereas NR_027151, AK056988 and uc003yqb.1 were down-regulated in nineteen pairs of HCC samples compared with adjacent NT samples. Moreover, expression of seven lncRNAs was significantly correlated to their nearby coding genes, suggesting these lncRNAs may play roles in HCC cells by regulating their nearby coding genes. For these dysregulated lncRNAs, we carried out lncRNA classification and subgroup analysis, and constructed lncRNA-mRNA co-expression network. Such information would be used for investigating the functions of these lncRNA in the occurrence and development of HCC. Our results revealed a penetrating layer of lncRNA of biological significance in pathologies of HCC, thereby pointing intriguing directions for further research.

## Supporting Information

Figure S1 **Results of RNA quantity and quality.**
(PDF)

Figure S2 **LncRNA-mRNA co-expression network.** 249 pairs of co-expressed lncRNAs and mRNAs composed of 131 mRNAs and 103 lncRNAs, and 146 pairs presented as a positive correlation. Pearson correlation coefficients between all aberrant lncRNAs and mRNAs were calculated, p-value <0.001 and absolute value of correlation coefficient ≥0.99. Red ellipse represents as down-regulated lncRNA, green diamond represents as down-regulated mRNA, blue ellipse represents as up-regulated lncRNA, black diamond represents as up-regulated mRNA. Solid lines represent positive correlation, dotted lines represent negative correlation.
(PDF)

Table S1 **Primers used for qRT-PCR.**
(XLSX)

Table S2 **Common Differentially Expressed mRNAs and lncRNAs in three HCC samples.**
(XLSX)

Table S3 **HOX cluster profiling.**
(XLSX)

Table S4 **Enhancer LncRNAs profiling.**
(XLSX)

Table S5 **Enhancer LncRNAs nearby coding gene data.**
(XLSX)

Table S6 **Rinn lincRNAs profiling.**
(XLS)

Table S7 **LincRNAs nearby coding gene data.**
(XLS)

Table S8 **Functional classification of mRNAs by GO.**
(XLSX)

Table S9 **△Ct values of lncRNAs in nineteen pairs of HCC samples and p value.**
(XLSX)

Table S10 **Seven differentially expressed lncRNAs and their nearby coding genes.**
(XLSX)
