# Supplementary material for: The Long Noncoding RNA Expression Profile of Hepatocellular Carcinoma Identified by Microarray Analysis
Source: PLoS One. 2014 Jul 15;9(7):e101707. doi: 10.1371/journal.pone.0101707 (PMC4099127; doi:10.1371/journal.pone.0101707)
Supplement: Figure S1 — Results of RNA quantity and quality. (PDF) [file pone.0101707.s001.pdf]

RNA QC

1. RNA Quantification and Quality Assurance by NanoDrop ND-1000

Sample ID	OD260/280 Ratio	OD260/230 Ratio	Conc.(ng/μl)	Volume(μl)	Quantity(ng)	QC result Pass or Fail
H1T	2.04	2.3	1280.15	160	204824	pass
H1N	2	2.26	2555.97	50	127798.5	pass
H2T	1.99	1.96	2965.32	50	148266	pass
H2N	2	2.06	1312.43	80	104994.4	pass
H3T	2.01	2.25	2560.67	30	76820.1	pass
H3N	2	2.13	1852.22	40	74088.8	pass

*For spectrophotometer, the O.D. A260/A280 ratio should be close to 2.0 for pure RNA (ratios between 1.8 and 2.1 are acceptable). The O.D. A260/A230 ratio should be more than 1.8.

2. RNA Integrity and gDNA contamination test by Denaturing Agarose Gel Electrophoresis

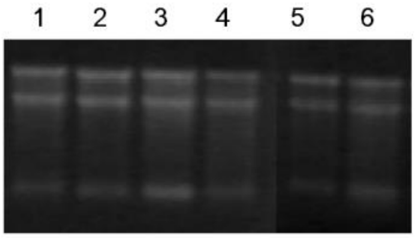

Lane 1: Total RNA of sample H1T

Lane 2: Total RNA of sample H1N

Lane 3: Total RNA of sample H2T

Lane 4: Total RNA of sample H2N

Lane 5: Total RNA of sample H3T

Lane 6: Total RNA of sample H3N

*The 28S and 18S ribosomal RNA bands should be fairly sharp, intense bands. The intensity of the upper band should be about twice that of the lower band. Smaller, more diffuse bands representing low molecular weight RNAs (tRNA and 5S ribosomal RNA) may be present. It is normal to see a diffuse smear of ethidium bromide stanting material migrating between the 18S and 28S ribosomal bands, probably comprised of mRNA and other heterogeneous RNA species. DNA contamination of the RNA preparation will be evident as a high molecular weight smear or band migrating above the 28S ribosomal RNA band. Degradation of the RNA will be reflected by smearing of ribosomal RNA bands.
