# Supplementary figures and images for: The Long Noncoding RNA Expression Profile of Hepatocellular Carcinoma Identified by Microarray Analysis
Source: PLoS One. 2014 Jul 15;9(7):e101707. doi: 10.1371/journal.pone.0101707 (PMC4099127; doi:10.1371/journal.pone.0101707)

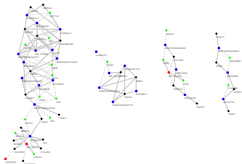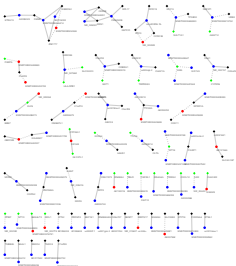

Supplement: Figure S2 — LncRNA-mRNA co-expression network. 249 pairs of co-expressed lncRNAs and mRNAs composed of 131 mRNAs and 103 lncRNAs, and 146 pairs presented as a positive correlation. Pearson correlation coefficients between all aberrant lncRNAs and mRNAs were calculated, p-value <0.001 and absolute value of correlation coefficient ≥0.99. Red ellipse represents as down-regulated lncRNA, green diamond represents as down-regulated mRNA, blue ellipse represents as up-regulated lncRNA, black diamond represents as up-regulated mRNA. Solid lines represent positive correlation, dotted lines represent negative correlation. (PDF) [file pone.0101707.s002.pdf]
